# Triticale Improvement for Forage and Cover Crop Uses in the Southern Great Plains of the United States

**DOI:** 10.3389/fpls.2018.01130

**Published:** 2018-08-06

**Authors:** Habtamu Ayalew, Tadele T. Kumssa, Twain J. Butler, Xue-Feng Ma

**Affiliations:** Noble Research Institute, Ardmore, OK, United States

**Keywords:** cover crop, crop improvement, forage, genomics, triticale

## Abstract

Triticale (×*Triticosecale* Wittmack) is a man-made species developed by crossing wheat (*Triticum* spp.) and rye (*Secale cereale* L.). It incorporates favorable alleles from both progenitor species (wheat and rye), enabling adaptation to environments that are less favorable for wheat yet providing better biomass yield and forage quality. Triticale has huge potential for both grain and forage production, though research to improve the crop for better adaptation and grain quality is lagging behind that of other small grains. It is also gaining popularity as a cover crop to improve soil health and reduce nutrient leaching. Because of its genetic and flower structure, triticale is suitable for both line and hybrid breeding methods. Advances in the areas of molecular biology and the wealth of genomic resources from both wheat and rye can be exploited for triticale improvement. Gene mapping and genomic selection will facilitate triticale breeding by increasing selection precision and reducing time and cost. The objectives of this review are to summarize current triticale production status, breeding, and genetics research achievements and to highlight gaps for future research.

## Introduction

Triticale (×*Triticosecale* Wittmack) is a man-made species developed by crossing wheat (*Triticum* spp.) and rye (*Secale cereale* L.). The first triticale, which was infertile, was developed in 1875 in Scotland (Stace, 1987). Later, in 1888, Rimpau crossed hexaploid wheat and rye to develop the first viable hybrid through spontaneous chromosome doubling in Germany (Mergoum et al., 2009). European countries were pioneers in both creating this very important species and breeding the new crop to adapt to various environments. The first improved commercial cultivar was released in Hungary in 1968 (Blum, 2014). Triticale breeding in North America was formally started in 1954 at the University of Manitoba in Canada, from which the first commercial variety, Rosner, was released in 1969 (Larter et al., 1970). During the mid-1960s, the University of Manitoba started a collaborative research program with CIMMYT (the International Maize and Wheat Improvement Center), and it is now the largest triticale breeding institute in the North America (Mergoum et al., 2009). In the United States, though there were attempts to develop triticale from wheat and rye hybridization starting in the early 1880s^1^, the formal breeding and production of triticale as a crop was started late in the last century.

Triticale is a hardy crop with prolific growth and adaptation to various environmental conditions. It combines the hardiness and nutrient-use efficiency of rye and high grain yield and nutritional qualities of wheat (Furman et al., 1997; Dennett et al., 2013). Improved triticale cultivars produce greater biomass and grain yield than wheat (Mergoum and Macpherson, 2004) and are comparable to rye (Kavanagh and Hall, 2015). This makes triticale a viable alternative crop especially in nutrient-deficient environments with various biotic and abiotic stress factors (Jessop, 1996; Blum, 2014; Randhawa et al., 2015; Liu et al., 2017).

Global triticale acreage covered over 4 million hectares of land with an average annual production of nearly 17 million tons of grain in 2014 (FAOSTAT, 2014). According to the same report, Poland, Germany, Belarus, and France were the leading triticale-producing countries, accounting for 72% of global production. Germany had the highest productivity followed by France, Poland, and Belarus (**Table 1**). Triticale acreage expanded by 8% in 2014, while total grain production increased by 17% compared with the previous year (FAOSTAT, 2014).

**Table 1:** Total area (hectare), average production (tons), and productivity (tons/ha) of the top 10 triticale growing countries in the world (FAOSTAT, 2014).

	Country	Hectare	Tons	Productivity (tons/ha)
1	Poland	1,306,025	5,246,647	4.02
2	Belarus	523,413	2,076,376	3.97
3	Germany	418,200	2,972,200	7.11
4	France	387,604	2,023,275	5.22
5	Russian Federation	247,553	654,135	2.64
6	China	212,000	385,000	1.82
7	Spain	195,682	449,674	1.82
8	Hungary	123,160	486,450	2.30
9	Lithuania	120,100	395,200	3.95
10	Australia	79,879	125,641	3.29

In the United States, triticale was mainly grown for animal feed (Blount et al., 2017b). It has only recently become popular as a forage and cover crop. The Southern Great Plains and the West Coast constitute a large proportion of the country’s triticale acreage. Despite the fact that triticale has huge forage and cover crop potential in the United States, currently it is only a minor crop covering a small percentage of cereals acreage. Relative to other small grains, triticale breeding and production have received limited attention (Blum, 2014) and funding because of the crop’s low grain-processing qualities for human consumption and high seed price (Blount et al., 2017a). In the United States, triticale growers were not covered by crop insurance until recently (USDA, 2017), which had made it even harder to compete with other cereals. However, the better performance of triticale, even in a less optimal environment (Blum, 2014; Kavanagh and Hall, 2015), gives it a competitive advantage over wheat and other alternative cool-season forage and cover crops.

Triticale has a large canopy cover that helps it intercept more sunlight, and it has strong and profuse roots that enable better soil anchorage. It has high nitrogen-acquisition capabilities and use efficiency, which makes it an ideal crop to grow after others have left much nitrogen in the soil (Long et al., 2013; Ketterings et al., 2015). It can also be grown between high N-crop cycles as a cover crop, after corn, to reduce N-leaching, and to control weeds (Mergoum et al., 2009; Ketterings et al., 2015).

Since triticale has wheat (diploid, tetraploid, and hexaploid), rye and the different forms (primary, secondary, and substituted) of triticale as its genetic resource base, genetic variation can continuously be created (through crossing) to enrich the genetic pool (Furman et al., 1997). However, the optimum utilization of triticale depends upon the accurate design and exploitation of germplasm through the applications of various breeding and genetics tools to stack desirable gene combinations in the crop. Breeding to develop an improved forage and cover crop will benefit from both conventional and molecular breeding tools. In this review, we aim to summarize the potential of triticale as an alternative forage and cover crop in the Southern Great Plains and the breeding and genetic improvement activities to date and to discuss the future prospects of triticale improvement, emphasizing forage and cover crop production.

## Genetic Architecture and Diversity

Triticale is an amphiploid cereal with several genome compositions depending on the type of wheat parent involved in the hybridization. Triticale can have ploidy levels ranging from tetraploid (2*n* = 14 = AARR) (Łapiński, 2002; McGoverin et al., 2011) to octoploid (2*n* = 56 = AABBDDRR) (Oettler et al., 1991; Furman et al., 1997; Oettler, 2005). Though octoploid triticale was developed first and extensively studied, it was not as adapted and productive as hexaploid triticale (Oettler, 2005). The most commonly cultivated type of triticale is hexaploid (2*n* = 42 = AABBRR), which has better adaptation and genomic stability than octoploid triticale (Ammar et al., 2004; Oettler, 2005). The primary hexaploid triticale is developed mainly by crossing durum wheat (*T. durum* Desf.) as a seed-bearing parent with rye as a pollen parent. The reverse order of parents, i.e., rye as a female and wheat as a male parent, did not produce a viable hybrid (Furman et al., 1997; Kavanagh and Hall, 2015). Octoploid triticale is less common because of its unstable genome and floral infertility (Mergoum et al., 2009). Hexaploidy was suggested to be the optimum genome size for triticale (Kiss, 1966). Interestingly, a cross between two octoploids results in hexaploid progeny (Oettler, 2005).

## Triticale Germplasm

Triticale can be classified as primary, secondary, and substituted types depending on the crossing subjects involved and the proportion of the progenitors’ genome finally retained in the new hybrid (Furman et al., 1997). Primary triticales are the direct amphidiploids of wheat and rye, while secondary triticales involve the intercrossing among primaries themselves or primaries with wheat in various combinations (Oettler, 2005; Mergoum et al., 2009; Hao et al., 2013). Through continuous breeding and intermating, some of the rye genomes are preferentially substituted by the D genome of wheat, resulting in genotypes with variable amounts of rye genome retained in substituted triticale (Gustafson et al., 1989; Furman et al., 1997; Hao et al., 2013). Triticale can also be classified as spring, winter and facultative types depending on vernalization requirements (Mergoum et al., 2009; McGoverin et al., 2011).

Secondary triticale is the most common and frequently cultivated form. Triticale developed from the cross between synthetic hexaploid wheat (AABBDD) and rye (RR) was reported to be a better alternative to incorporate valuable genetic variation through preferential partial substitution of the R genome by the D genome of *Aegilops tauschii* Coss. (Ammar et al., 2004; Hao et al., 2013). Incorporating fragments of the D genome from *Ae.* *tauschii* into hexaploid triticale through synthetic wheat–rye crosses and subsequent selection for deletion or substitution of hexaploid lines provides valuable genetic variation for plant vigor (ter Steege et al., 2005) and grain quality (McGoverin et al., 2011). Most triticale cultivars were developed from secondary triticale gene pools by line breeding. As triticale is a synthetic species, it has a short history of natural evolution, so there is a need to create, preserve, and exchange genetic materials between different institutions. Generally, triticale has a very narrow genetic base, as most of its germplasm was developed from only a few advanced materials that were intercrossed. Crossing between advanced/improved wheat and rye cultivars will create a better genetic combination to exploit the genetic gains from the breeding efforts of both parental species. Continuous screening and evaluation of these valuable genetic stocks for target sets of environments and traits of interest is required to fine-tune the importance of all the crossing endeavors (Furman et al., 1997).

## Triticale Forage Status in the Southern Great Plains

The Southern Great Plains region is characterized by stocker cattle production and widespread production of small grains for forage (Ball et al., 2007). Small grains including rye, wheat, triticale, and oat are the most commonly grown forage crops in the region, especially during the autumn and winter seasons (Newell and Butler, 2013; Kim et al., 2017). Because of increased corn silage prices, producers are shifting toward growing small grains to fill the forage gap during fall-winter seasons (Marsalis et al., 2008; Blount et al., 2017b). Triticale is emerging as a potential alternative to wheat and rye in the region because of its winter hardiness and high protein quality for animal feed (Furman et al., 1997; Dennett et al., 2013). In a comparative study of three small grains (wheat, rye, and triticale) conducted in the southern Oklahoma, triticale showed consistently better forage yield than wheat and was on par with rye (Kim et al., 2017). In a similar experiment conducted in the northern Mexico, winter and facultative triticale significantly outperformed both wheat and rye for dry matter yield (Hede, 2000). It also provides quality silage material to cover the forage gap during dry, hot summers (Delogu et al., 2002). Stalcup (2009) reported, quoting Brent Bean from the Texas A&M AgriLife Extension, that triticale showed 25% greater silage yield than wheat grown under a fully irrigation system.

In addition to high productivity (biomass and grain), triticale has good protein content and essential amino acids (lysine) composition. Compared with wheat, triticale showed higher protein content (10–20% on a dry weight basis) but similar amino acid composition, except for lysine (triticale was better) (Mergoum et al., 2009). Because of its higher starch digestibility, triticale is a better ruminant feed than other cereals. It is a strategic crop that combines the merits of both wheat and rye to exploit for winter forage in the Southern Great Plains.

## Potential of Triticale as a Cover Crop in the Southern Great Plains

Plants selectively grown to reduce soil erosion, enhance water infiltration, control weeds and pests, and improve soil health are generally termed cover crops. Growing cover crops after the main-season harvest is now becoming a common practice throughout the United States. Despite the widespread use of cover crops nationally, reports on economic benefits are mixed. Some writers have voiced concerns about the ecological and economic benefits of cover crops, especially in terms of water usage (Robinson and Nielsen, 2015; Nielsen et al., 2016). According to Nielsen and Vigil (2010), soil moisture was reduced by 20%, resulting in a 25% yield reduction in the subsequent wheat crop, when the conventional 14 months wheat-fallow tillage production system was replaced by 2.5 months of legume cover crop. On the other hand, in a more recent study, Adhikari et al. (2017) reported that a winter wheat cover crop did not cause any significant reduction in either soil water or cotton seed yield in the Texas Rolling Plains. According to the annual survey report from the Conservation Technology Information Center (CTIC, 2017), grain yields of wheat and soybean increased by 3 and 4%, respectively, compared with fallow precursors. However, there seems to be a delicate balance between the planting and termination of cover crops for subsequent cropping, which causes significant interactions between location, management and environment. Most growers in the region give greater emphasis on improving soil health even in seasons when economic return might not be satisfactory, which makes the prospect of cover crops promising.

The seemingly conflicting reports may be due to environmental and agronomic specificity. Benefits from cover crops can be maximized with the right seed selection and agronomic management that suits the target of producers. Though moisture deficit is the main limiting factor in the Southern Great Plains, the use of cover crops can still be economically justified with proper management and termination timing. Cover crops can also be grazed before the next commercial crops are planted for direct economic return. Rye has been popular among cover crop growers because of its weed-smothering capacity and high carbon residue (CTIC, 2017). In this regard, triticale, as a rye progeny, can be a viable alternative as a cover crop. Triticale is among the best overwintering species that help reduce soil erosion and capture residual nitrogen, which in turn increases annual forage yield and quality (Ketterings et al., 2015). Autumn-sown triticale showed 27 kg/ha nitrogen uptake, which was comparable to that of rye (32 kg/ha) in the Northeast United States (Ort et al., 2013). In a similar report, triticale showed a range of 23–34 kg/ha N uptake in the western New York (Long et al., 2013).

A separate crop ideotype that suits the edaphic and atmospheric scenarios of target environments will help optimize gains from cover crops. High allelopathy is one of the potential traits to look for in triticale and rye breeding for better weed control (Cheng and Cheng, 2015). To date, none of the triticale varieties used as cover crops was purposely selected for cover crop use. Vigorous and high-yielding triticale varieties that are required for high winter forage may not be good options as cover crops in the Southern Great Plains, as they may cause water and nutrient depletion for the next crop. Therefore, it will be reasonable to have selection traits tailored for cover crop purpose only.

## Conventional Triticale Breeding and Improvement

### Pure Line Selection

Triticale is a self-pollinating species with a low degree of out-crossing; as a result, it is amenable for pure line selection. Pure line selection involves the hybridization of two or more parents and segregation of lines until they attain homozygosity before any selection is practiced except for some qualitative traits that are easy to score (Lelley, 2006; Randhawa et al., 2015). However, in the case of triticale, selection in segregating lines needs to be delayed until the wheat–rye genome composition attains stability (Lelley, 2006).

Though the first wheat–rye hybrid was developed about 140 years ago (Stace, 1987), formal triticale breeding and selection was started in the mid-1950s (Larter et al., 1970). A decade later, in the mid-1960s, the University of Manitoba in Canada started a collaborative research program with CIMMYT in Mexico, forming the largest triticale breeding institute in North America (Mergoum et al., 2009).

The main challenges at the start of these breeding programs were excessive plant height and lodging, flower sterility, delayed maturity, and shriveled grains (Mergoum et al., 2009). Through coordinated breeding efforts, improved varieties better adapted to various environments have been released since then (Supplementary Table 1). The accidental identification of a triticale naturally out-crossed with a semi-dwarf bread wheat resulted in the first major breakthrough in triticale breeding (Hede, 2000). Grain yield was improved from 2.4 to 10 t/ha (>300% increase), plant height was reduced by 20 cm on average, and grain test weight was improved from 65.8 to 72 kg/hl (Hede, 2000).

Triticale breeding was focused mainly on improving grain yield for human consumption (Randhawa et al., 2015). However, the initial grain cultivars were spring-type, which contributed to their inconsistent performance and lower popularity in the market (Randhawa et al., 2015; Blount et al., 2017b). The competitive market prices of other crops that were covered under crop insurance were also one of the reasons why triticale was not as popular among growers as first expected (Blount et al., 2017a).

Triticale is gaining popularity as a winter forage crop. The Central Great Plains and West Coast regions of the United States produce a substantial amount of triticale annually (Blount et al., 2017b). Research to develop high-yielding and winter-hardy triticale cultivars is a priority at the Noble Research Institute to fill the forage gap during winter-fall seasons (Newell and Butler, 2013). Past research endeavors showed a 20% improvement in triticale productivity for forage yield (Saha et al., 2015).

Though forage and grain yield are the main agronomic traits of selection in triticale breeding, other component traits also need to be improved to achieve market adoption. Lodging, pre-harvest sprouting, disease susceptibility and inferior grain end-use quality are still unsolved challenges in triticale breeding (Sodkiewicz, 2002; Tyrka and Chelkowski, 2004; Randhawa et al., 2015). Contrary to obvious expectations, triticale is not as winter-hardy as rye because of genetic inhibition of the rye freezing-tolerance gene by the wheat genome (Blum, 2014). Therefore, breeding for winter hardiness needs to incorporate more freezing-tolerance genes from wheat until the genetic barrier inhibiting rye genes is resolved through research. Improving the end-use quality of triticale will mark the beginning of a new era in the evolution of the crop.

Future breeding research for forage and cover crop use needs to focus on generating genotypes that are well-adapted to cold winters and have high biomass yield between successive cuttings. Improving seedling early vigor will enable faster crop establishment and early ground cover, enhancing soil health and nutrient use efficiency (Salmon et al., 2004; Casler and Van Santen, 2010; Ayalew et al., 2018).

### Hybrid Breeding and Heterosis

Current triticale breeding is mainly dependent on developing inbred cultivars. Hybrid breeding can also be easily applied in triticale, as inbred parent development and seed multiplication mechanisms are easier than with other small grains. Triticale is a self-pollinating species, enabling easy inbred parent development with low inbreeding depression. In addition, triticale inherits cytoplasmic male sterility (CMS) from its wheat parent and the male fertility restorer gene from its rye background, enabling efficient hybrid seed multiplication (Góral et al., 2015). Pollen grains from a viable male parent can pollinate male sterile lines (seed parents) to a distance of 3–4 m away from the pollen source, ensuring ample pollen grains for hybrid seed production (Góral, 2004). As a result, there has been a growing interest among breeders in developing hybrid triticale cultivars (Barker and Varughese, 1992; Warzecha et al., 2014; Góral et al., 2015). Previous studies reported vigorous vegetative growth and high grain yield in triticale hybrids because of non-additive gene actions (Barker and Varughese, 1992; Oettler et al., 2005). Average mid-parent heterosis values ranging from 8.6 to 10.3% over mid-parent were reported for grain yield (Oettler et al., 2005; Fischer et al., 2010). Rye contributes high levels of non-additive genetic variability to the triticale genome (Oettler et al., 1991), which makes hybrid triticale breeding promising and justified. Developing viable progeny from a rye–wheat cross, with rye as a female parent, may also open a new source of genetic variability for stress tolerance in triticale. So far, rye as a female parent has not produced fertile progeny (Furman et al., 1997; Kavanagh and Hall, 2015). Grain and biomass yields are controlled by high levels of dominant gene actions (specific combining ability), while additive gene effects (general combining ability) control other yield component traits (Oettler et al., 2005). Therefore, with careful selection of parental lines that have high general and specific combining abilities, hybrid breeding provides a viable option to exploit heterosis for forage and grain yield.

## Molecular Breeding Applications in Triticale

### Molecular Markers and Linkage Mapping

Molecular markers play a major role in the genetic improvement of crop plants by facilitating the identification and tagging of important genes for potential transfer or cloning (Semagn et al., 2006; Collard and Mackill, 2008). Marker-assisted selection (MAS) is one of the oldest applications of marker technologies in plant breeding. Preferred markers in MAS are those that are abundant in the genome, polymorphic even within closely related individuals, reproducible and amenable for automation (Semagn et al., 2006; Collard and Mackill, 2008; Beyene et al., 2016). Functional markers (markers in the gene itself) are the best types of markers in MAS, as they are completely linked to the quantitative trait loci (QTL) or gene (zero chance of crossover) (Varshney et al., 2005, 2014). However, these kinds of markers are not as abundant as other marker types, which are mostly from the non-coding regions of the genome. Genotyping by sequencing (GBS) is becoming the standard genotyping technology in terms of its throughput and ease of developing simple nucleotide polymorphism (SNP) markers (Poland et al., 2012).

Triticale genomics can benefit from marker developments and genomics tools in both wheat and rye, as large proportions of the two parental genomes are conserved in triticale (Ma et al., 2004; Ma and Gustafson, 2008). However, triticale is not a mere mix of the rye and wheat genomes. Allopolyploidization of the two genomes results in sequence modifications or losses of 10–30% in wheat and up to 50% in rye genomes (Boyko et al., 1984; Ma et al., 2004; Ma and Gustafson, 2008). As a result, wheat and rye markers may not be fully informative in triticale.

Unlike wheat and rye, only a limited number of markers have been directly developed from triticale itself (Kuleung et al., 2004; Badea et al., 2011). Most of the markers used in triticale were developed from either wheat or rye. However, transferability of the limited number of markers tried so far was low. According to Kuleung et al. (2004), only 39% of rye and 57% of wheat simple sequence repeat (SSR) markers were transferable to triticale, indicating the need to screen more markers (types) to get a working marker density in triticale. Transferability of markers seemed also to depend on the type of marker technology used. Badea et al. (2011) reported lower polymorphic information content (PIC) of diversity array technology (DArT) markers originating from either wheat or rye genomes than was previously reported for SSR markers (Kuleung et al., 2004).

Returns from prior investments in wheat and rye genome sequencing can be exploited through comparative genomics and mapping of triticale with wheat and rye, thereby equipping the genomic toolbox of triticale. Comparative mapping of barley with wheat (Close et al., 2009) and switchgrass with foxtail millet (Daverdin et al., 2015) were reported to be useful in the genomic developments of the respective crops.

The first triticale linkage map had only 356 markers on 73 double haploid (DH) lines (González et al., 2005), which had neither enough marker density nor even distribution within and between chromosomes. A fairly dense map (one unique locus every 4 cM) was reported by Tyrka et al. (2011), though most of the markers still tended to be on the R genome. Incorporation of more marker types (SSR, DArT, and DArTSeq markers) improved the resolution to one marker every 3 cM density (Tyrka et al., 2015). A consensus map consisting of 2,555 DArT markers spanning a distance of 2,309.9 cM with an average marker density of one unique locus every 1.2 cM showed the highest resolution and genome coverage (Alheit et al., 2011). However, this consensus map did not have uniform marker distribution among the three genomes either. Marker saturation in triticale depends on the contrast between parental lines and diversity in the mapping population (Tyrka et al., 2015), which necessitates constructing a consensus map that incorporates markers from several populations. Most triticale genetic maps were based on DH populations, which reduced the genetic variability that could have been created through meiotic crossing-over in subsequent generations. Incorporating SNPs will improve genome coverage and precision of QTL or gene localization.

### Quantitative Trait Loci (QTLs) Mapping

For successful application of markers in plant breeding, tight marker-trait linkage or association is essential. Having informative markers and dense genetic maps alone does not guarantee successful QTL mapping. The appropriate number of mapping individuals and the nature of mapping population (structured or unstructured) are also important factors to consider for accurate detection of QTLs. The optimum mapping population size is dependent on the nature of segregation of markers and traits in the mapping population (Bogdan and Doerge, 2005; Li et al., 2010). Generally, the number of detected QTLs increases with increasing population size (Schön et al., 2004; Li et al., 2010).

Several previous studies in triticale reported identification of QTLs for various traits, such as biomass yield, grain yield, thousand-kernel weight, and plant height (Busemeyer et al., 2013; Alheit et al., 2014; Würschum et al., 2014; Liu et al., 2016, 2017). However, most previous studies did not validate markers and QTLs for MAS, a common drawback in many QTL mapping studies. QTLs that were reported using bi-parental populations (DH and RIL) might not be valid on genetic backgrounds other than the mapping populations themselves because such populations hardly represent the available diversity in the germplasm.

Grain and biomass yield, biotic and abiotic stress tolerance, and grain and forage quality traits are controlled by many QTLs. This makes QTL mapping difficult especially with the small number of genotypes. Information generated using bi-parental mapping needs to be validated before any further investment to use it in MAS. Many major-effect QTLs were identified and validated for root architecture under water stress in wheat (Ayalew et al., 2017) and rice (Uga et al., 2013), and disease resistance in wheat (Ma, 2010).

Quantitative trait loci can also be identified and mapped using transcript variations of mapping lines as substitutes for phenotype data to find the marker-transcript association termed expression QTLs (eQTLs) (Schadt et al., 2003). This technique could be especially important in triticale to understand the biological and genetic processes happening in the development of the hybrid by revealing the gene regulatory network of the whole genome. Expression QTLs were reported in several species, including barley, soybean, and rice (Chen et al., 2010; Bolon et al., 2014; Wang et al., 2014; Kuroha et al., 2017), unraveling gene regulatory networks.

Bi-parental QTL mapping approach was instrumental in understanding the genetic mechanisms of different traits. However, applications of research outputs (QTLs and linked markers) in MAS were far less than satisfactory because of several limitations in the approach (Heffner et al., 2009). Low accuracy in QTL size and location; low representation of genomic allele distribution; and, as a result, the need for validation of markers and QTLs for MAS hampered translation of genetic gains into practical plant breeding (Jannink et al., 2001; Heffner et al., 2009). In addition to inflated QTL effects and imprecise genomic locations of conventionally mapped QTLs, results generated from bi-parental mapping populations hardly represent the allelic distribution of a trait in a species (Heffner et al., 2009; Talukder and Saha, 2017).

Linkage disequilibrium (LD)-based QTL mapping was proposed to avoid these limitations. Markers are in LD when they are consistently co-inherited without being chromosomally linked (Slatkin, 2008). This approach helps avoid the need to develop structured mapping populations, and it utilizes LD instead of genetic linkage to dissect the genetics of complex traits in breeding populations (Jannink et al., 2001). As the marker-trait association is rather genome-wide, it is called genome-wide association study.

In triticale, though most genotypes originate from a limited number of crosses (Ammar et al., 2004), chromosomal substitutions, inversions and rearrangements create linkage between non-collinear loci (LD). Significant LD among the three genomes and population structures has been reported in triticale (Alheit et al., 2012). Growth habits were identified as the main sources of population structure, with the R genome being the less diverse genome compared with the other two (A and B) genomes (Alheit et al., 2012).

Benefits from QTL mapping and MAS will be realized only when the marker-QTL linkage or association is close enough to avoid breakage during meiosis and when the identified QTLs have significant phenotypic effects. Apart from the limitations of conventional MAS in practical crop improvement (Heffner et al., 2009), most previous studies on triticale are mere reports of QTL identification that need further effort to validate the effects of the identified QTLs and amenability of flanking markers for MAS.

## Future Breeding Strategies

The success of any breeding program lies in the creation, acquisition, and proper phenotyping of diverse germplasm for target traits and environments. There is a tremendous possibility of generating genetic variability in triticale through the various parental combinations and the random chromosome reshuffling during meiosis and genomic changes after allopolyploidization.

Triticale breeding especially for forage and cover crop use needs to incorporate high grain and biomass yield and grazing tolerance, quick rejuvenation after successive cuttings or grazing, and high disease resistance. Unlocking the inhibited rye freezing-tolerance gene in triticale will tremendously improve freezing tolerance in triticale, thereby making it more fit for the winter grazing system in many parts of the United States. The selection of genotypes with high early vigor will enable early ground cover and efficient resource utilization, thereby improving both soil health and forage production.

Improving nitrogen use efficiency will help fit triticale in rotation cropping after maize, thereby making use of the excess N leftover from the previous crop cycle. Improving lodging will also enable intensive farm input application with a high rate of return.

High-throughput field phenotyping coupled with fast greenhouse or growth chamber screening methods that corroborate field-grown data need to be developed to feed new germplasm into the breeding pipeline to hasten the breeding process.

Pure-line selection will remain the mainstay of breeding in triticale because of both the efficiency of pure-line cultivars and the low market interest for hybrid cultivars. However, with the incorporation of appropriate hybridization techniques for easy seed production, lower production costs and efforts to tackle some of the existing triticale quality problems, hybrid triticale will also have the potential to exploit heterosis. The best chromosomal arrangement or combination in hybrids is yet to be determined for the various target traits and environments.

Though there was promise from the advances in molecular genetics, much effort is needed when it comes to triticale improvement. The mere identification of QTLs will not take us any further unless the identified QTLs are validated and the flanking markers are used in real-life application. Modern genomic tools have to be incorporated into the current breeding strategy to facilitate the precision and efficiency of selection. MAS need to be practiced in triticale with the appropriate number and types of markers to select individuals or genome regions that influence the genetic expression and inheritance of target traits. Though we could not find reports of genomic selection (GS) in triticale, it has been reported that estimating the genetic value of individuals is a more effective strategy than using individual marker loci to tag genome regions that influence a trait of interest. Combining GS with noble phenotyping techniques at an early stage will hasten the breeding process, thereby improving genetic gain per cycle of selection.

## Author Contributions

HA and X-FM drafted and finalized the manuscript. TK and TB reviewed the manuscript.

## Conflict of Interest Statement

The authors declare that the research was conducted in the absence of any commercial or financial relationships that could be construed as a potential conflict of interest.

## Footnotes

1 http://www.triticale-infos.eu
