# Supplementary material for: Triticale Improvement for Forage and Cover Crop Uses in the Southern Great Plains of the United States
Source: Front Plant Sci. 2018 Aug 6;9:1130. doi: 10.3389/fpls.2018.01130 (PMC6087761; doi:10.3389/fpls.2018.01130)
Supplement: Supplementary file 1 [file Table_1.DOCX]

**Supplementary Table 1.** **Partial list of triticale varieties registered in the USA during 1975-2017 (USDA-AMS-ST-PVPO, Washington, DC).**

| PVP No. | Variety/Name | Applicant | Status Date |
| --- | --- | --- | --- |
| 7400067 | 6TA205 | Jenkins Foundation for Research | 4/15/1975 |
| 7100013 | 6TA-385 | Triticale Breeders, Inc. | 6/10/1977 |
| 7300087 | 6TA418 | Jenkins Foundation for Research | 9/13/1977 |
| 7300088 | 6TA419 | Jenkins Foundation for Research | 9/13/1977 |
| 7900096 | Councill | Alabama A&M University | 9/17/1980 |
| 7100012 | 6TA-203 | Triticale Breeders, Inc. | 9/24/1982 |
| 7100014 | 6TA-204 | Triticale Breeders, Inc. | 9/24/1982 |
| 7100015 | 6TA 131 | Triticale Breeders, Inc. | 9/24/1982 |
| 9300197 | Plains | Agrecol Corporation | 12/26/1996 |
| 9600010 | 301 | Resource Seeds, Inc. | 9/21/2000 |
| 9700391 | 762 | Northern Agri Brands, LLC | 10/27/2000 |
| 8200032 | Grace | Resource Seeds, Inc. | 6/30/2001 |
| 8100001 | Jenkins | Resource Seeds, Inc. | 1/27/2002 |
| 8200026 | Nutricale | Fred C. Elliott | 1/27/2002 |
| 9800375 | 815 | Northern Agri Brands, LLC | 6/10/2002 |
| 9900400 | 105 | Northern Agri Brands, LLC | 6/10/2002 |
| 9900401 | 111 | Northern Agri Brands, LLC | 6/10/2002 |
| 200100051 | Alzo | Plant Breeding and Acclimatization Institute | 9/20/2002 |
| 200200022 | Bobcat | Alberta Agriculture, Food & Rural Development Field Crop Development Centre | 9/20/2002 |
| 9600361 | 498 | Northern Agri Brands, LLC | 1/30/2003 |
| 200200215 | 348 | Northern Agri Brands, LLC | 1/30/2003 |
| 200200216 | 336 | Northern Agri Brands, LLC | 1/30/2003 |
| 200200267 | 2115 | Northern Agri Brands, LLC | 4/29/2003 |
| 200200268 | 308 | Northern Agri Brands, LLC | 4/29/2003 |
| 200200269 | 2205 | Northern Agri Brands, LLC | 4/29/2003 |
| 200200153 | NE422T | Board of Regents, University of Nebraska | 8/27/2004 |
| 200600007 | TAMcale 5019 | Texas Agricultural Experiment Station | 2/14/2006 |
| 200600008 | TAMcale 6331 | Texas Agricultural Experiment Station | 2/14/2006 |
| 200300338 | 346 | Northern Agri Brands, LLC | 6/5/2006 |
| 200300339 | 1029S | Northern Agri Brands, LLC | 6/5/2006 |
| 200400001 | 314 | Northern Agri Brands, LLC | 6/5/2006 |
| 200400002 | 96 | Northern Agri Brands, LLC | 6/5/2006 |
| 200400161 | Forerunner | Weaver Seed of Oregon Inc. & Oregon Trail Seeds Inc. | 6/5/2006 |
| 200500049 | Monarch | Florida Agricultural Experiment Station (FAES) University of Georgia Research Foundation, Inc. (UGARF) | 6/9/2006 |
| 200600020 | 342 | Florida Agricultural Experiment Station and University of Georgia Research Foundation | 2/6/2007 |
| 200600303 | 98 | Northern Agri Brands, LLC | 9/7/2007 |
| 200600304 | 116 | Northern Agri Brands, LLC | 9/7/2007 |
| 8400144 | Triti-Gold 22 | Dean Bork | 9/29/2007 |
| 8700205 | Stan-I | Resource Seeds, Inc. | 4/30/2008 |
| 8700206 | Eve | Resource Seeds, Inc. | 4/30/2008 |
| 8700207 | Victoria | Resource Seeds, Inc. | 4/30/2008 |
| 200800406 | Pacheco | Dr. Peter Franck | 4/1/2009 |
| 200800401 | 888 | Northern Agri Brands, LLC | 5/5/2009 |
| 200900277 | Pacheco | Dr. Peter Franck | 8/7/2009 |
| 200900444 | 718 | Northern Agri Brands, LLC | 11/22/2010 |
| 201100152 | 718S | Northern Agri Brands, LLC | 8/14/2012 |
| 9100054 | Stan II | Resource Seeds, Inc. | 10/31/2012 |
| 9200279 | Enduro | Agrecol Corporation | 10/31/2012 |
| 9300122 | 2700 | Resource Seeds, Inc. | 11/30/2012 |
| 201200082 | SY 115T | Northern Agri Brands, LLC | 6/25/2014 |
| 201200083 | SY 158T | Northern Agri Brands, LLC | 6/25/2014 |
| 201200084 | 141 | Northern Agri Brands, LLC | 6/25/2014 |
| 201200468 | HyOctane | Dr. Peter Franck | 6/25/2014 |
| 201300439 | Fredro | Danko Hodowla Roslin Sp 200 | 9/11/2014 |
| 201400328 | SY TF 131 | Northern Agri Brands, LLC | 3/30/2015 |
| 201400329 | SY TF 813 | Northern Agri Brands, LLC | 3/30/2015 |
| 9300259 | Norico | Drs. Fred C. and Nancy A. Elliott | 8/31/2015 |
| 9300306 | Buxom | Drs. Fred C. and Nancy A. Elliott | 8/31/2015 |
| 201400033 | Traction | DR. PETER FRANCK | 9/29/2015 |
| 9100032 | Roughrider | Goertzen Seed Research | 10/31/2015 |
| 9500316 | Blizzard | Drs. Fred C. and Nancy A. Elliott | 12/29/2015 |
| 9400023 | 6600 | Northern Agri Brands, LLC | 2/29/2016 |
| 201500218 | NC01PT-1433 | North Carolina State University | 5/12/2016 |
| 201500374 | SY TF 135 | Northern Agri Brands, LLC | 6/3/2016 |
| 9400201 | 102 | Northern Agri Brands, LLC | 6/28/2016 |
| 9300303 | Companion | Drs. Fred C. and Nancy A. Elliott | 7/31/2016 |
| 201700108 | Circuit | DR. PETER FRANCK | 2/24/2017 |
| 201600408 | NF201 | The Samuel Roberts Noble Foundation, Inc. | 5/23/2017 |
| 201500222 | Wheatly | Dr. Nancy A. Elliott | 6/27/2017 |
| 201500505 | Short Beard Thunder | Board of Regents, University of Nebraska | 6/27/2017 |
| 201600410 | 946802617 | Northern Agri Brands, LLC | 10/4/2017 |
| 201700268 | NT11428 | Board of Regents, University of Nebraska | 10/4/2017 |
| 201700269 | NT11406 | Board of Regents, University of Nebraska | 10/4/2017 |
| 201700386 | 618491724 | Northern Agri Brands, LLC | 10/4/2017 |
| 201700387 | 641512175 | Northern Agri Brands, LLC | 10/4/2017 |
| 201700388 | 261216487 | Northern Agri Brands, LLC | 10/4/2017 |
| 201700389 | 841446398 | Northern Agri Brands, LLC | 10/4/2017 |
